# Comparative transcriptome analyses revealed differential strategies of roots and leaves from methyl jasmonate treatment *Baphicacanthus cusia* (Nees) Bremek and differentially expressed genes involved in tryptophan biosynthesis

**DOI:** 10.1371/journal.pone.0212863

**Published:** 2019-03-13

**Authors:** Wenjin Lin, Wei Huang, Shuju Ning, Xiaogui Gong, Qi Ye, Daozhi Wei

**Affiliations:** 1 School of Life science, Fujian Agriculture and Forestry University, Fuzhou, Fujian, China; 2 Fujian Key Laboratory of Medical Measurement, Fujian Academy of Medical Sciences, Fuzhou, Fujian, China; 3 School of Crop science, Fujian Agriculture and Forestry University, Fuzhou, Fujian, China; ICAR-Indian Institute of Agricultural Biotechnology, INDIA

## Abstract

*Baphicacanthus cusia* (Nees) Bremek (*B*. *cusia*) is an effective herb for the treatment of acute promyelocytic leukemia and psoriasis in traditional Chinese medicine. Methyl jasmonate (MeJA) is a well-known signaling phytohormone that triggers gene expression in secondary metabolism. Currently, MeJA-mediated biosynthesis of indigo and indirubin in *B*. *cusia* is not well understood. In this study, we analyzed the content of indigo and indirubin in leaf and root tissues of *B*. *cusia* with high-performance liquid chromatography and measured photosynthetic characteristics of leaves treated by MeJA using FluorCam6 Fluorometer and chlorophyll fluorescence using the portable photosynthesis system CIRAS-2. We performed de novo RNA-seq of *B*. *cusia* leaf and root transcriptional profiles to investigate differentially expressed genes (DEGs) in response to exogenous MeJA application. The amount of indigo in MeJA-treated leaves were higher than that in controled leaves (*p* = 0.004), and the amounts of indigo in treated roots was higher than that in controlled roots (*p* = 0.048); Chlorophyll fluorescence of leaves treated with MeJA were significantly decreased. Leaves treated with MeJA showed lower photosynthetic rate compared to the control in the absence of MeJA. Functional annotation of DEGs showed the DEGs related to growth and development processes were down-regulated in the treated leaves, while most of the unigenes involved in the defense response were up-regulated in treated roots. This coincided with the effects of MeJA on photosynthetic characteristics and chlorophyll fluorescence. The qRT-PCR results showed that MeJA appears to down-regulate the gene expression of tryptophan synthase β-subunits (trpA-β) in leaves but increased the gene expression of anthranilate synthase (trp 3) in roots responsible for increased indigo content. The results showed that MeJA suppressed leaf photosynthesis for *B*. *cusia* and this growth-defense trade-off may contribute to the improved adaptability of *B*. *cusia* in changing environments.

## Introduction

For plants to survive in nature, they need to make choices when faced with various biotic or abiotic stresses in their surrounding environments, resulting in the production of chemical defenses [1, 2]. In response to biotic or abiotic stresses, plants employ alternative tolerance or resistance strategies to protect themselves against various internal and external signals [3]. Jasmonates (JAs) -mediated induced resistance is an important mechanism of phytochemical defense [4, 5]. JAs are important plant hormones that are necessary for plant growth and development [6], stress resistance [7, 8], secondary metabolism [9, 10] and cell cycle regulation [11]. JAs mainly include cyclopentanone derivatives, such as jasmonic acid, methyl jasmonate, isoleucine jasmonate and 12-oxo-phytodienoic acid [12]. Previous genome-wide transcriptome profile analysis discovered that treating plants with JAs such as MeJA can induce extensive transcriptional alterations via the biosynthesis of terpenoids [13–15], phenylpropanoids [16–18], alkaloids [19–21] and volatile organic compounds [22, 23].

*Baphicacanthus cusia* (Nees) Bremek (*B*. *cusia*) is generally distributed in southern China, Bangladesh, northeast India, Myanmar, Himalayan and the mid-south Peninsula [24]. The root and aerial parts of *B*. *cusia* are used as medicinal materials in Nan-Ban-Lan-Gen [25] and Indigo Naturalis [26], respectively. These were widely used as traditional Chinese medicine to remove heat from blood and eliminate toxicity in the human body [27]. Pharmacological studies have shown that Nan-Ban-Lan-Gen has many biological activities, such as antibacterial [28], antiviral [29, 30], immunomodulatory [31, 32] and anti-inflammatory activities [33]. Previous clinical studies indicated that Indigo Naturalis is good for the treatment of acute promyelocytic leukemia [34, 35], ulcerative colitis [36, 37], and psoriatic lesions [38]. And that the secondary metabolites, such as indirubin, indigo and tryptanthrin were the active components [39]. The molecular mechanism of the production of the active components *B*. *cusia* in response to biotic or abiotic stresses has not been reported.

In our previous study [40], tryptophan synthase was confirmed to be the candidate gene involved in biosynthesis of indican, which was one of the genes in the tryptophan biosynthesis pathway. Hence, we speculate that the key genes affecting the biosynthesis of indigo and indirubin are the genes involved in the tryptophan biosynthesis pathway, the upstream pathway for the biosynthesis of indican.

In this study, to obtain in-depth knowledge of indican biosynthesis upstream gene expression changes in MeJA-treated leaves and roots, we performed de novo high-throughput sequencing of *B*. *cusia* leaves and roots before and after MeJA treatment. The assembled unigenes were annotated by five databases: nr, SwissProt, GO, COG and KEGG. We focused on the differentially expressed genes (DEGs) in the MeJA-treated *B*. *cusia* leaves and roots. Furthermore, we identified several candidate genes associated with indican biosynthesis via the upstream tryptophan pathway by qRT-PCR. Meanwhile, we determined the content of indigo and indirubin in leaf and root tissues of *B*. *cusia* and measured the photosynthetic characteristics and chlorophyll fluorescence of leaves treated by MeJA. This is the first report on the transcriptional response of *B*. *cusia* leaves and roots treated by MeJA. The molecular mechanisms underlying MeJA treatment will promote research on the biological mechanisms involved in molecular breeding and secondary metabolite regulation of *B*. *cusia*. The transcriptome may help to clarify differentiated strategies of roots and leaves in response to exogenous application of methyl jasmonate in *B*. *cusia*.

## Results

### RNA-seq, de novo assembly and unigene annotation

The RNA-seq of twelve cDNA libraries of leaf and root samples produced approximately 36 G bases of total nucleotides. After removing the low-quality reads adapters and more than 10% of unknown nucleotides, there were 280,702,858 high-quality reads obtained from roots and 267,021,714 from leaves. A total of 51,381 unigenes were generated with an N50 of 1932 bp, an average length of 1232 bp and a GC percentage of 42.42%. The number of genes expressed was 44,858 (87.30%) for CL, 44,951 (87.49%) for TL, 48,497 (94.39%) for CR, and 47,634 (92.71%) for TR. A total of 33,317 annotated unigenes (64.84% of all unigenes) were obtained. Among them, 32,898 (98.74%) were matched in nr, 25,809 (77.46%) in Swiss-Prot, 20,751 (62.28%) in KOG, and 13,232 (39.72%) in KEGG.

### DEGs in response to MeJA

**Fig 1:**
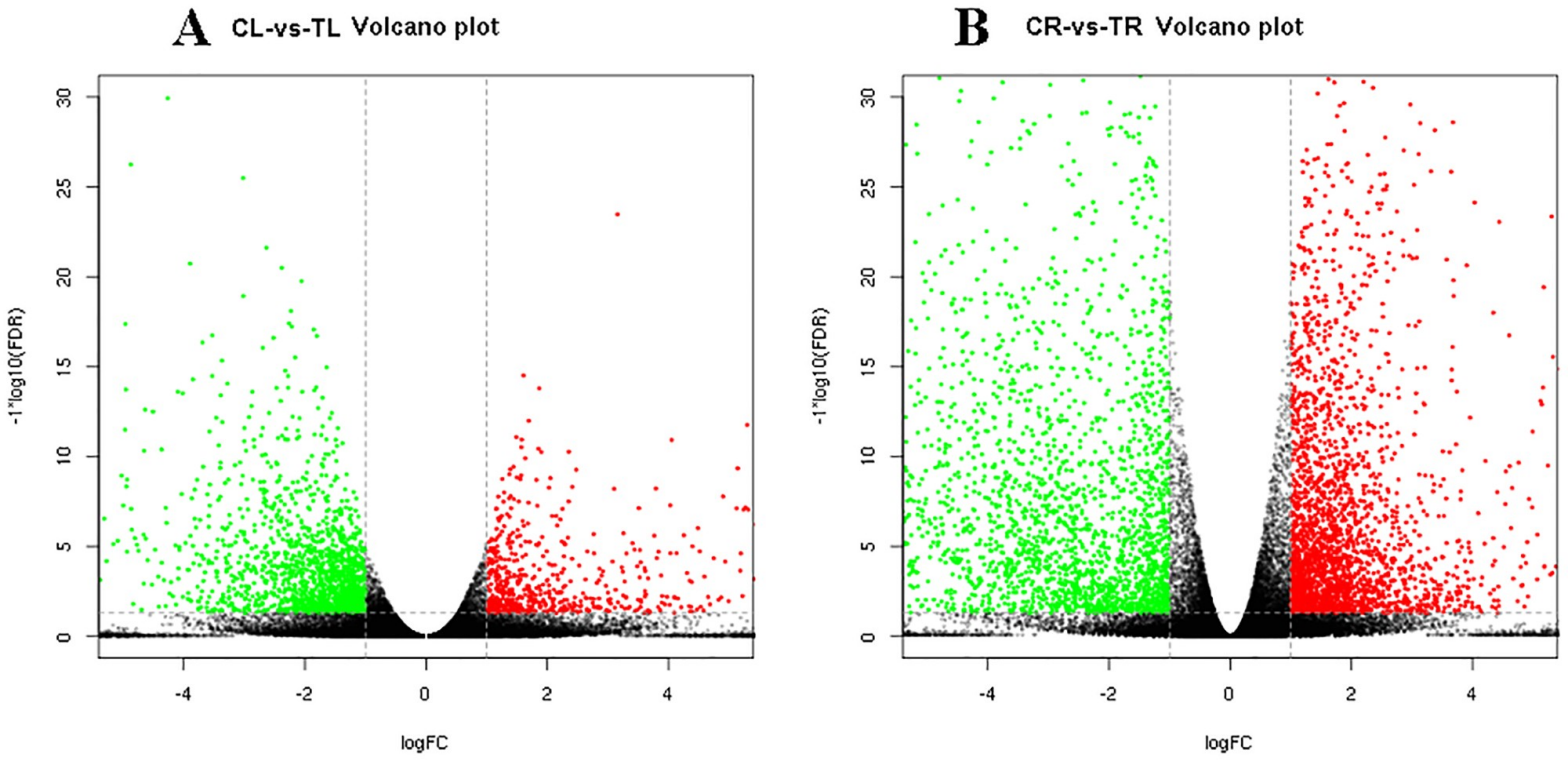
Volcano plots of up-regulated and down-regulated DEGs of leaves and roots from methyl jasmonate treated *B*. *cusia*. (A) Volcano plots of CL-VS-TL; (B) Volcano plots of CR-VS-TR, The red (Treated sample is up-regulated relative to controlled sample) and green (down-regulated) points indicate differences in gene expression (threshold of fold change >2 and false discovery rate <0.05), and the black dots indicate no difference between the two samples. The abscissa indicates the log of the fold change and the ordinate indicates the negative Log10 value of the FDR.

In response to MeJA treatment, 8,355 DEGs were found to be significantly differentially expressed in 33,317 annotated unigenes, among which 2,664 DEGs were up-regulated and 3,335 DEGs were down-regulated in MeJA-treated roots and 761 DEGs were up-regulated and 1,595 DEGs were down-regulated in MeJA-treated leaves (Fig 1). These results suggest that leaves of *B*. *cusia* demonstrate greater suppression of unigenes than activation in response to MeJA treatment; in contrast, roots of *B*. *cusia* demonstrate greater activation than suppression of unigenes in response to MeJA treament.

### GO functional annotation of DEGs

**Fig 2:**
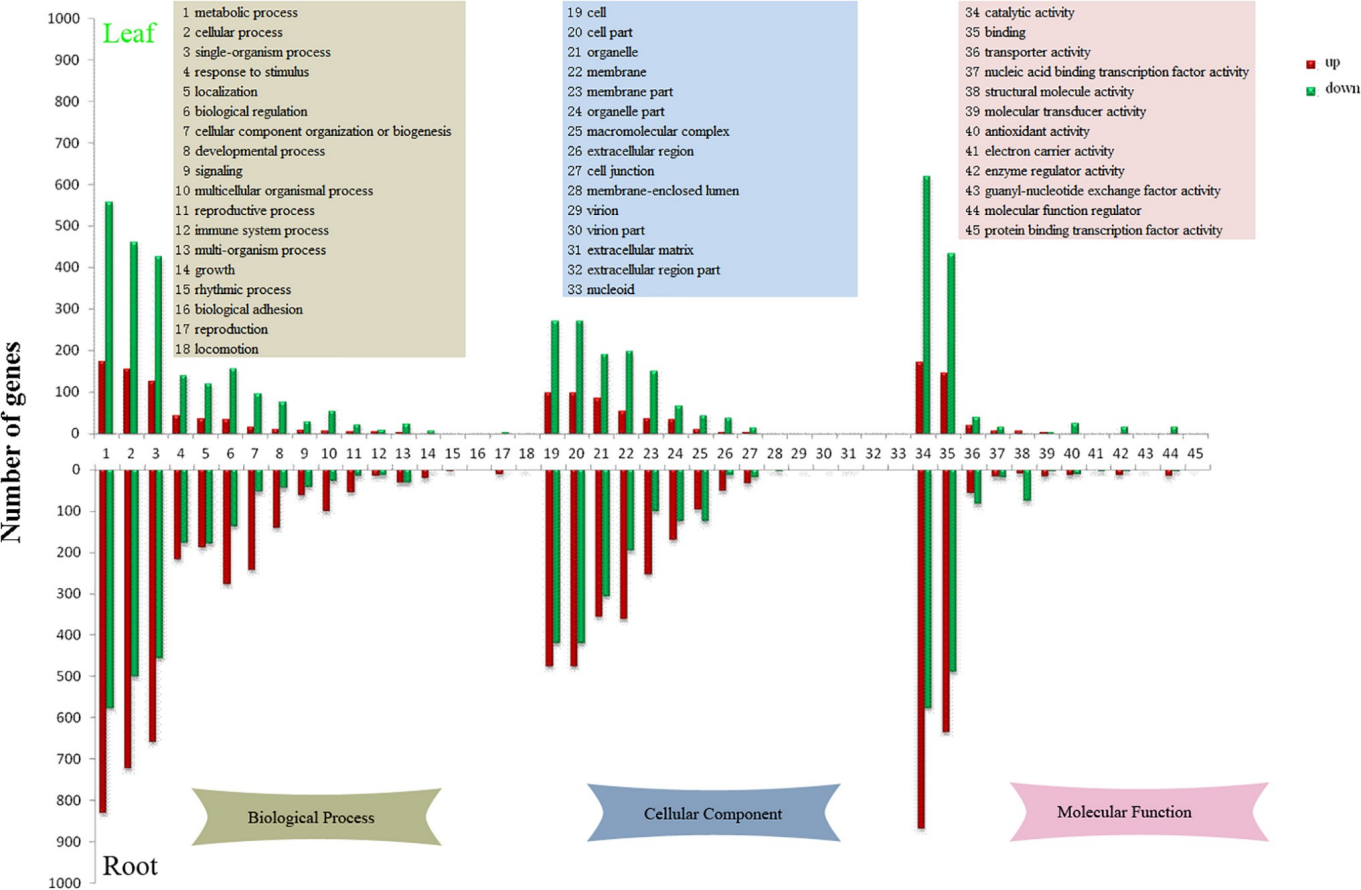
Histogram of level 2 GO terms of CL-VS-TL and CR-VS-TR. The upper part of the figure shows level 2 GO terms of CL-VS-TL, the lower part of the figure shows level 2 GO terms of CR-VS-TR. The red bars show up-regulated unigenes and green shows down-regulated unigenes. The yellow rectangle shows GO terms of biological process, the blue rectangle shows GO terms of cellular component, and the pink rectangle shows GO terms of molecular function.

We obtained GO function annotation based on the GO classification of annotated unigenes. 6,099 DEGs were categorized into 43 GO classes between CL and TL (S1 Table), among which the class of “catalytic activity” (793, 13.00%) was predominant. Moreover, a high proportion of unigenes were categorized as “metabolic process” (732, 12.00%), followed by “cellular process” (616, 10.10%) and “binding” (581, 9.53%). Only one gene was assigned to the classes “virion”, “virion part”, “protein binding transcription factor activity”, “nucleoid”, “extracellular region part” and “extracellular matrix”. A total of 12,842 unigenes were categorized to 42 GO functional classes between CR and TR (S2 Table), among which the class of “catalytic activity” (1443, 11.24%) was predominant. In addition, a high proportion of genes were categorized to “metabolic process” (1406, 10.95%) followed by “cellular process” (1224, 9.53%) and “binding” (1125, 8.76%). Only a few genes were assigned to the classes “locomotion” (3, 0.02%), “protein binding transcription factor activity” (3, 0.02%), “guanyl-nucleotide exchange factor activity” (3, 0.02%), “virion” (3, 0.02%) and “virion part” (3, 0.02%). The results of GO terms enrichment analysis of the *B*. *cusia* DEGs are listed in Fig 2. From the GO enrichment analysis, we can see that most of the GO terms were down-regulated significantly in CL-VS-TL and up-regulated significantly in CR-VS-TR.

### KEGG pathway classification of unigenes

To identify the specific biological pathways of unigenes assembled above, the KEGG pathway database was employed to characterize the functional classification and pathway mapping according to sequence homology. Overall, 744 of 11,418 unigenes were classified into five main categories and 107 KEGG pathways, which included cellular processes, environmental information processing, genetic information processing, metabolism and organismal systems, in leaves and 1,528 of 11,980 unigenes were classified into the same five main categories and 121 KEGG pathways in roots. As shown in Table 1, in CL-VS-TL (S3 Table), the carbohydrate metabolism has the highest number of unigenes (150), followed by amino acid metabolism (95), biosynthesis of other secondary metabolites (79), lipid metabolism (66), and global and overview (63). In CR-VS-TR (S4 Table), the carbohydrate metabolism category also has the highest number of unigenes (254), but is followed by translation (161) of genetic information processing, global and overview (150), amino acid metabolism (124), lipid metabolism (121), and energy metabolism (108).

**Table 1:** Distributions of unigenes in KEGG pathway database classification.

KEGG_A_class	KEGG_B_class	CL-VS-TL	CR-VS-TR
Cellular Processes	Transport and catabolism	14	77
Environmental Information Processing	Membrane transport	0	12
	Signal transduction	19	54
Genetic Information Processing	Folding, sorting and degradation	14	76
	Replication and repair	40	29
	Transcription	1	26
	Translation	13	161
Metabolism	Amino acid metabolism	95	124
	Biosynthesis of other secondary metabolites	79	79
	Carbohydrate metabolism	150	254
	Energy metabolism	40	108
	Global and Overview	63	150
	Glycan biosynthesis and metabolism	9	25
	Lipid metabolism	66	121
	Metabolism of cofactors and vitamins	24	41
	Metabolism of other amino acids	36	62
	Metabolism of terpenoids and polyketides	45	56
	Nucleotide metabolism	21	27
Organismal Systems	Environmental adaptation	15	46

### Analysis of KEGG pathway enrichment

We identified 196 and 73 DEGs remarkably enriched in fourteen and three KEGG pathways in CL-VS-TL and CR-VS-TR, respectively. In CL-VS-TL, the DEGs were mostly associated with phenylpropanoid biosynthesis (46), starch and sucrose metabolism (28), pentose and glucuronate interconversions (18), DNA replication (17) and terpenoid backbone biosynthesis (12) (Table 2). Most of the DEGs were down-regulated, such as photosystem I subunit IV (PsaE), photosystem I subunit VI (PsaH), photosystem I subunit X (PsaK), photosystem I subunit PsaN (PsaN), photosystem I subunit PsaO (PsaO) on ko00195 (S1 Fig); light-harvesting complex I chlorophyll a/b binding protein 2 (LHCA2), light-harvesting complex II chlorophyll a/b binding protein 1 (LHCB1), LHCB2, LHCB3, LHCB4, LHCB6 on ko00196 (S2 Fig), with the exception of flap endonuclease-1(FEN1) on ko03030, L-iditol 2-dehydrogenase (SORD) on ko00040, aldehyde dehydrogenase (ALDH) on ko00040 and ko00903, and endoglucanase on ko00500.

**Table 2:** KEGG pathway enrichment DEGs in leaves of MeJA-treated *B*.*cusi*a.

Pathway	Gene number	*p*-value	*q*-value	Pathway ID
Phenylpropanoid biosynthesis	204	3.75E-17	4.01E-15	ko00940
Photosynthesis—antenna proteins	19	2.27E-07	1.21E-05	ko00196
DNA replication	78	7.18E-07	2.56E-05	ko03030
Isoquinoline alkaloid biosynthesis	32	3.64E-05	9.74E-04	ko00950
Pentose and glucuronate interconversions	120	8.15E-05	1.75E-03	ko00040
Cutin, suberine and wax biosynthesis	37	0.000127	2.26E-03	ko00073
Ubiquinone and other terpenoid-quinone biosynthesis	60	0.000345	5.27E-03	ko00130
Sesquiterpenoid and triterpenoid biosynthesis	43	0.000428	5.73E-03	ko00909
Phenylalanine metabolism	65	0.000704	8.37E-03	ko00360
Stilbenoid, diarylheptanoid and gingerol biosynthesis	38	0.000856	9.16E-03	ko00945
Anthocyanin biosynthesis	10	0.001422	1.38E-02	ko00942
Terpenoid backbone biosynthesis	84	0.00191	1.70E-02	ko00900
Limonene and pinene degradation	18	0.00226	1.74E-02	ko00903
Starch and sucrose metabolism	290	0.00228	1.74E-02	ko00500

In CR-VS-TR, the only three KEGG pathways significantly enriched were sesquiterpenoid and triterpenoid biosynthesis (18, *p*-value 2.12E-06, *q*-value 0.000257), photosynthesis-antenna proteins (11, *p*-value 4.38E-06, *q*-value 0.000265), and phenylpropanoid biosynthesis (44, *p*-value 0.000330, *q*-value 0.013310). Interestingly, almost all DEGs in the three pathways were up-regulated, such as farnesyl-diphosphate farnesyltransferase, germacrene D synthase, vetispiradiene synthase on ko00909, photosystem II oxygen-evolving enhancer protein 1 (PsbO), PsbP, PsbQ, Psb27, PsaE, PsaG, PsaH, PsaK, PsaL, PsaN, PsaO, cytochrome b6-f complex iron-sulfur subunit (PetC), plastocyanin (PetE) on ko00195 (S3 Fig); light-harvesting complex I chlorophyll a/b binding protein 1 (LHCA1), LHCA2, LHCA3, LHCA4, light-harvesting complex II chlorophyll a/b binding protein 1 (LHCB1), LHCB2, LHCB3, LHCB6 on ko00196 (S4 Fig), β-glucosidase, cinnamoyl-CoA reductase, shikimate O-hydroxycinnamoyl- transferase and caffeoyl-CoA O-methyltransferase on ko00940.

**Fig 3:**
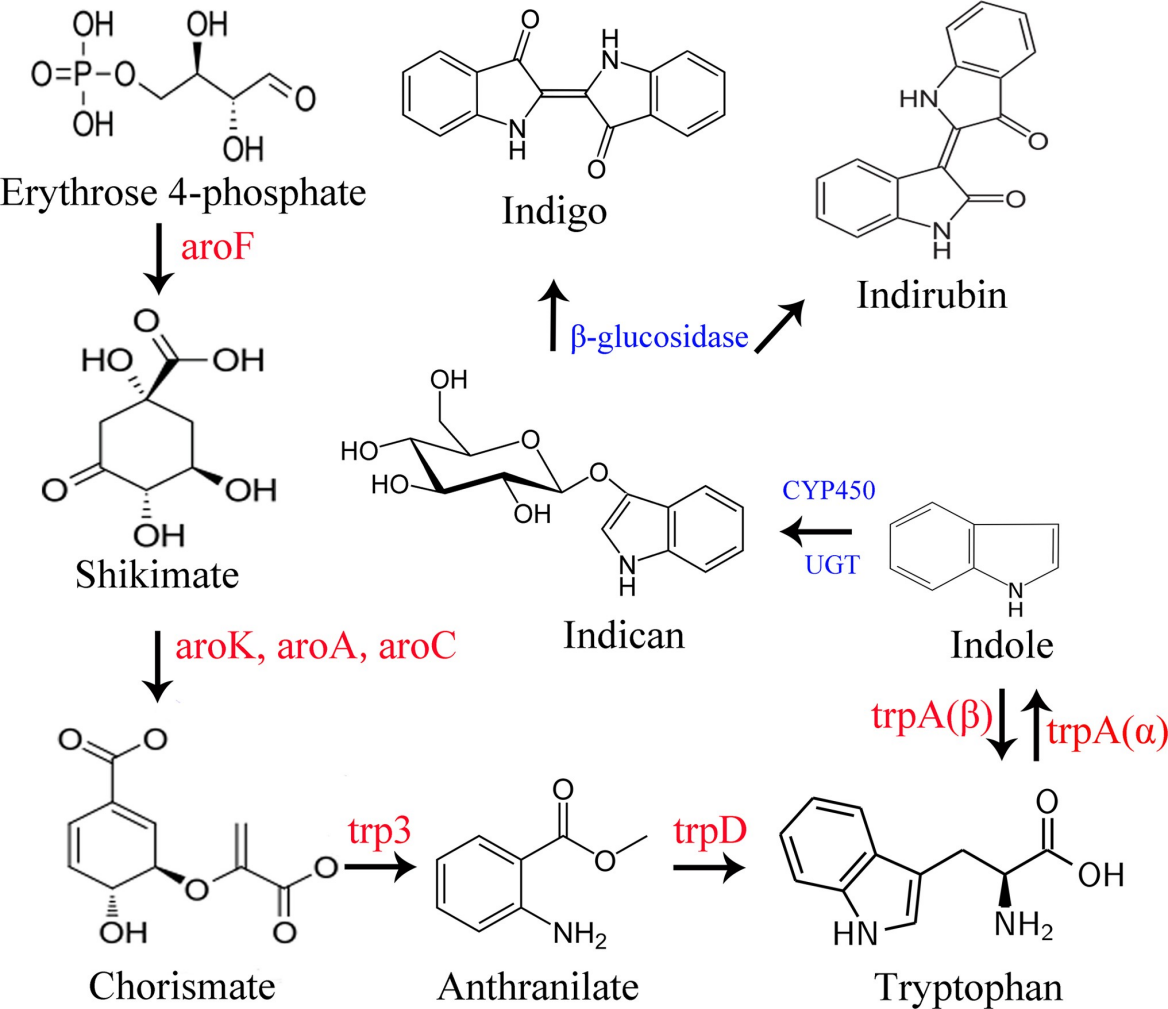
Pathway of phenylalanine, tyrosine and tryptophan biosynthesis and putative pathway of indican biosynthesis and metabolism. The red word in the figure shows the genes involved in tryptophan biosynthesis, the blue shows the genes involved in indican biosynthesis and metabolism. aroF: 3-deoxy-7-phosphoheptulonate synthase; aroK: shikimate kinase; aroA: 3-phosphoshikimate 1-carboxyvinyltransferase; aroC: chorismate synthase; trp3: anthranilate synthase; trpD: anthranilate phosphoribosyltransferase; trpA: tryptophan synthase.

Indican is the precursor of indigo and indirubin (Fig 3). For the purpose of understanding the biosynthesis pathway of indican in *B*. *cusia*, we found five and six DEGs enriched in phenylalanine, tyrosine and tryptophan biosynthesis pathways (ko00400) in CL-VS-TL and CR-VS-TR, respectively. In CL-VS-TL, all of the DEGs involved in phenylalanine, tyrosine and tryptophan biosynthesis were down-regulated, such as 3-deoxy-7-phosphoheptulonate synthase (aroF), 3-phosphoshikimate 1-carboxyvinyltransferase (aroA), arogenate dehydratase (ADT), prephenate dehydratase (pheA2), aspartate aminotransferase (GOT1), and arogenate dehydrogenase (tyrAa). In CR-VS-TR, the DEGs tyrosine aminotransferase (TAT), aroA and chorismate synthase (aroC) were down-regulated, but anthranilate synthase (TRP3) and tryptophan synthase alpha chain (trpA) were up-regulated.

### Validation of RNA-seq analysis by qRT-PCR

To verify the relative expression levels of DEGs involved in the biosynthesis of phenylalanine, tyrosine and tryptophan obtained by RNA sequencing, we carried out qRT-PCR on eight relative unigenes (aroF, aroK, aroA, aroC, TRP3, trpD, trpA-α and trpA-β) involved in the biosynthesis of tryptophan.

**Fig 4:**
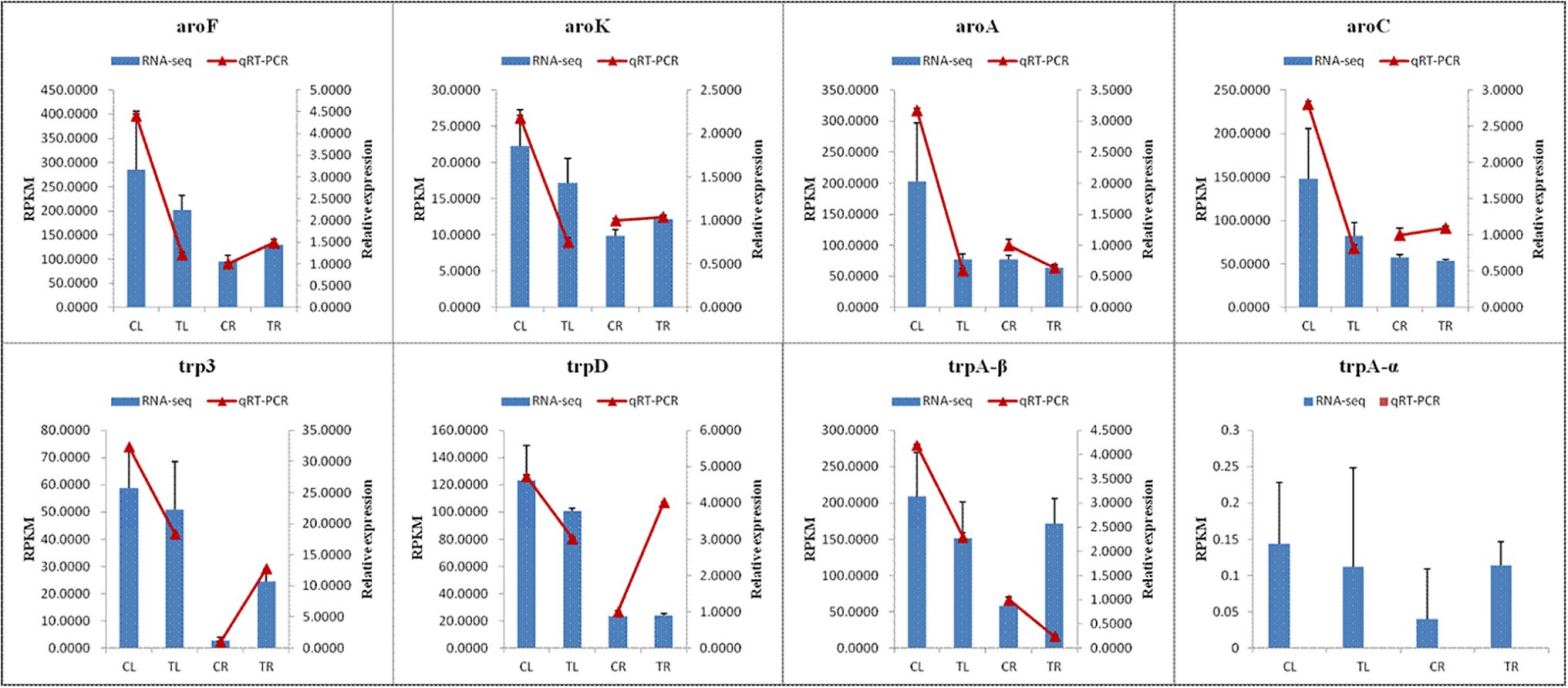
qRT-PCR validation of DEGs involved in tryptophan biosynthesis.

The results of qRT-PCR analysis revealed that the relative expression of candidate unigenes, which were down-regulated in CL-VS-TL, were consistent with the data of RNA-seq RPKM; in CR-VS-TR, the relative expression of all candidate unigenes was consistent with data of the RNA-seq RPKM except for trpA-β and aroC, the relative expression of trpA-β and aroC in CR-VS-TR were the opposite of the RNA-seq data (Fig 4), the relative expression of trpA-α have not detected.

### Quantitative analysis of indigo and indirubin

**Fig 5:**
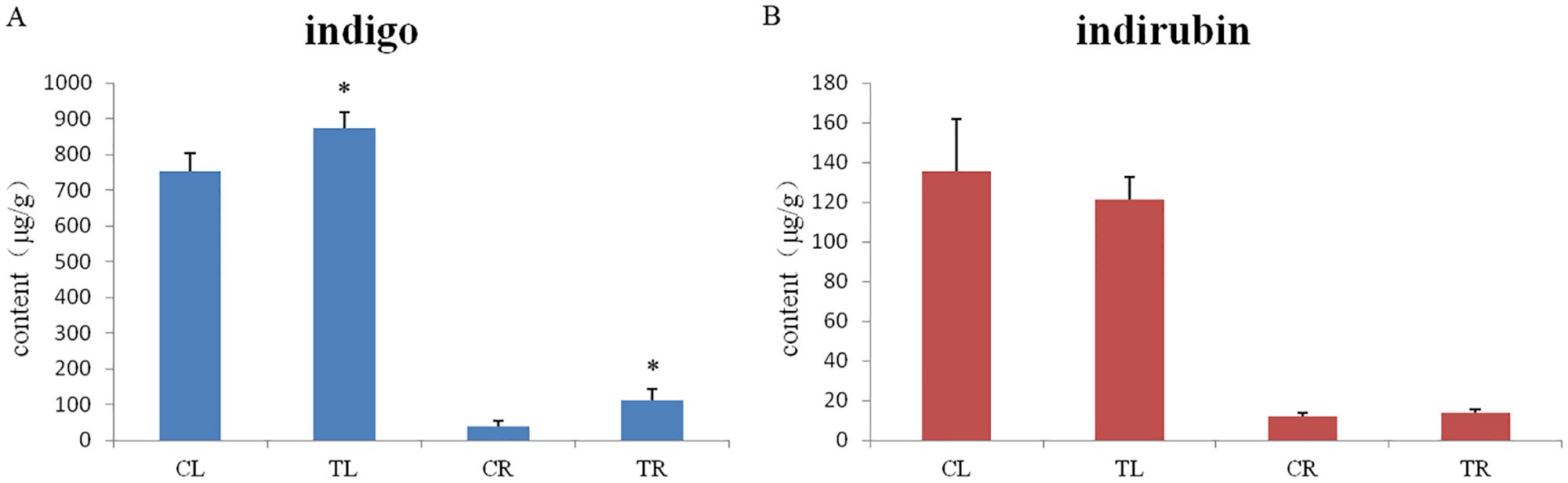
Contents of indigo and indirubin in leaf and root of *B*.*cusia*. (A) content of indigo; (B) content of indirubin.

The calibration curves for indigo and indirubin were prepared using five different concentration mix reference materials. The regression equations and correlation coefficients (r^2^) were: Y = 7.1499X + 3.6795 (linear range from 13.92 to 125.25μg∙L^-1^), r^2^ = 0.9996 for indigo; Y = 16.342X + 2.7178 (linear range from 9.53 to 85.79μg∙L^-1^), r^2^ = 0.9997 for indirubin. The relative amounts (μg/g) of indigo and indirubin in the leaf and root tissues were calculated using the above equations. The amounts of indigo in TL were higher than those in CL (*p* = 0.004, Fig 5), and the amounts of indigo in TR were higher than those in CR (*p* = 0.048); there were no differences in the amounts of indirubin between CL and TL (*p* = 0.273), as well as between CR and TR (*p* = 0.904).

### Influence of MeJA on appearance and traits of the leaves and roots

**Fig 6:**
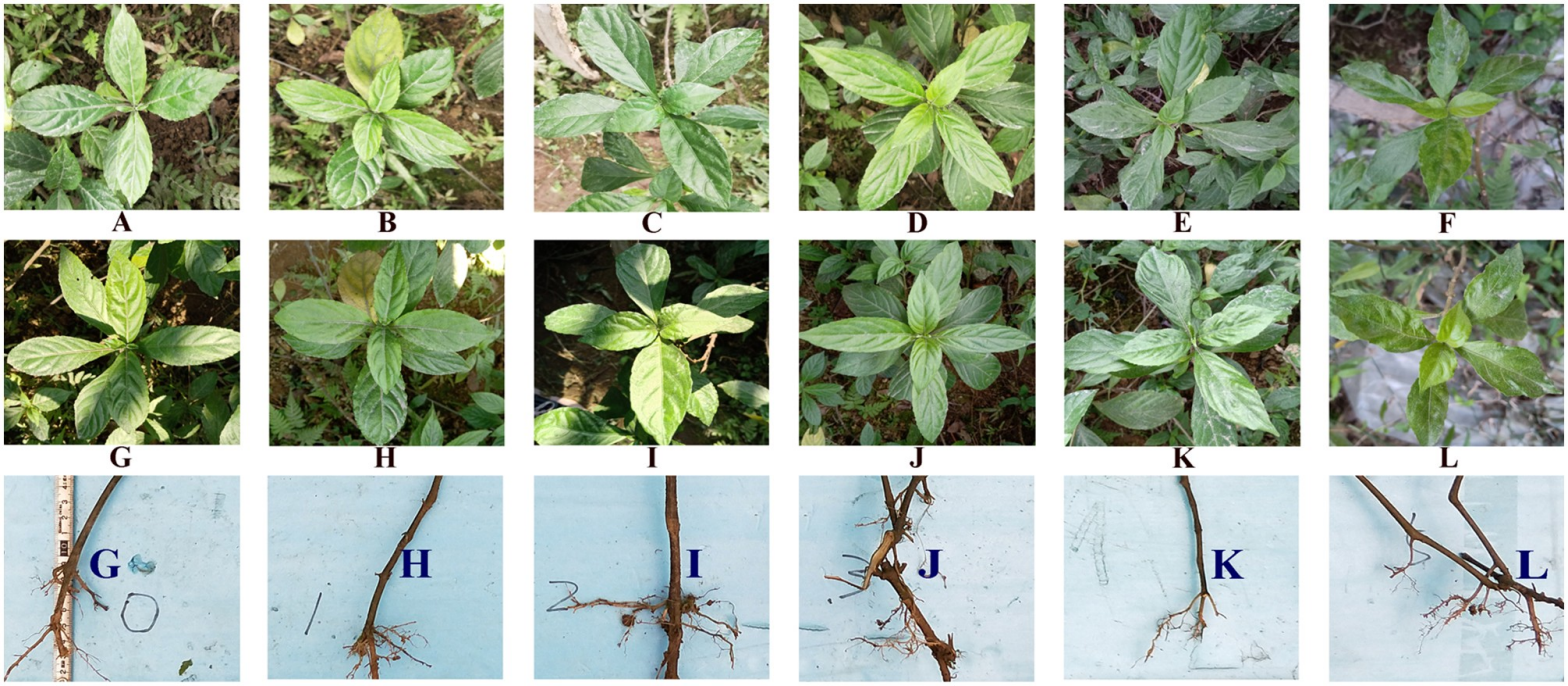
Effects of different concentrations of MeJA on *B*. *cusia* leaves and roots. A~F before treatment, G~L after treatment, G: Control groups, treated with a solution of 0.01% (v/v) Tween 20; H: Treated group 1, treated with a solution of 0.01% (v/v) Tween 20 containing 2,229 μM MeJA; I: Treated group 2, treated with a solution of 0.01% (v/v) Tween 20 containing 222.9 μM MeJA; J: Treated group 3, treated with a solution of 0.01% (v/v) Tween 20 containing 22.29 μM MeJA; K: Treated group 4, treated with a solution of 0.01% (v/v) Tween 20 containing 22.29 mM MeJA; L: Treated group 5, treated with a solution of 0.01% (v/v) Tween 20 containing 222.9 mM MeJA.

In this experiment, we have done the field trials of the effects of different concentrations of methyl jasmonate on *B*. *cusia* (22.29 μM, 222.9 μM, 2,229 μM, 22.29 mM and 222.9 mM). In addition to the control group, we set up five treatment groups with more than three biological samples per group (S5 Fig). The growth conditions for field trials (2019.1.1~2019.1.31 at Fuzhou) were showed in supplementary file (S6 Table), the data were downloaded from http://rp5.ru/archive.php?wmo_id=58847&lang=cn. The leaf area is calculated by the leaf length multiplied by 0.7 times the leaf width. The row data of length and width of *B*. *cusia* leaves was shown in supplementary file (S7 Table). The results indicated that the leaves showed different degrees of shrinkage after treated with methyl jasmonate (Fig 6, S6 Fig), the leaf area of the leaves of the control group increased by 47.44%, and leaf areas decreased by 12.98%, 22.15%, -6.93%, 22.12%, 24.68% after treated with 22.29 μM, 222.9 μM, 2,229 μM, 22.29 mM and 222.9 mM methyl jasmonate. Compared with the control group, the leaf area of each group decreased after treatment with methyl jasmonate (*P* = 0.000, 0.000, 0.004, 0.000, 0.000), and the roots showed more hairy roots after treatment with different concentrations of methyl jasmonate (Fig 6, S6 Fig). In the Fig 6, the number of hair roots in the control group was approximately 10, and 16, 18, 20, 14, 35 hair roots in the treatent groups, after treated with 22.29 μM, 222.9 μM, 2,229 μM, 22.29 mM and 222.9 mM methyl jasmonate.

### Influence of MeJA on photosynthetic parameters and chlorophyll fluorescence

**Fig 7:**
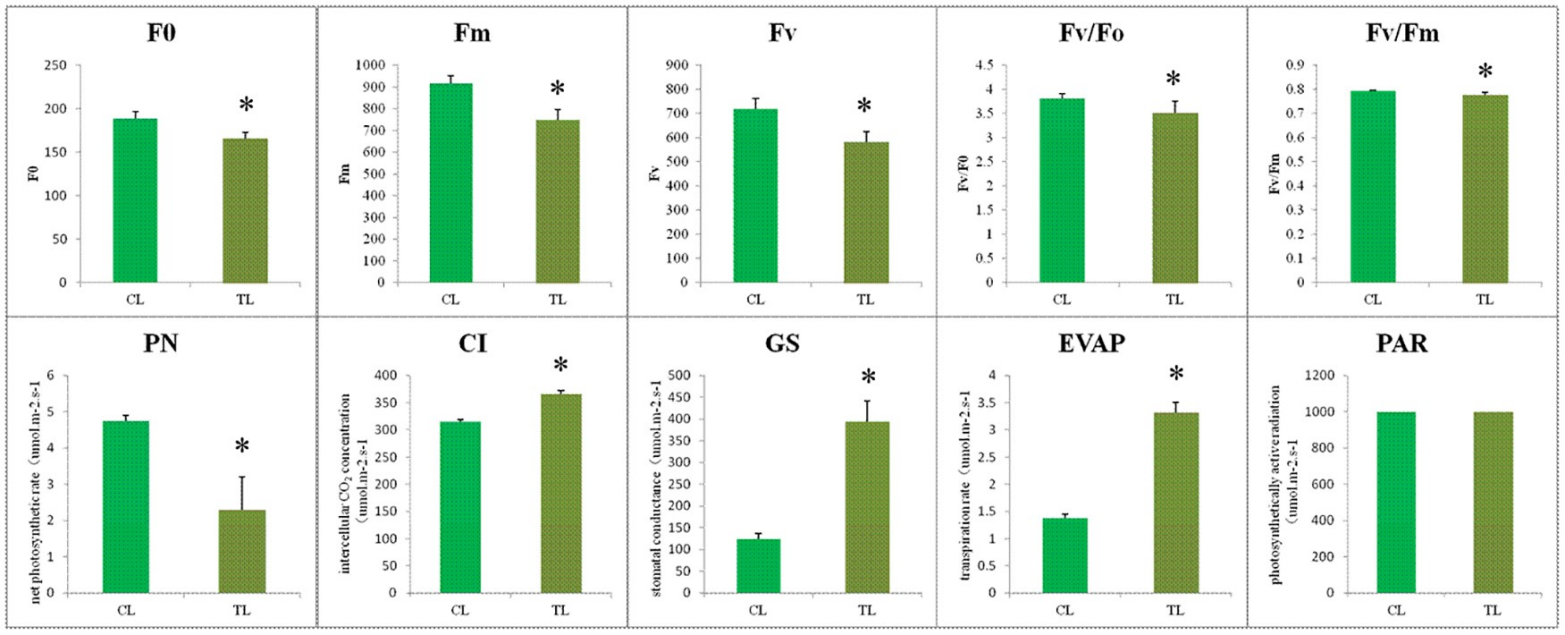
Leaf chlorophyll fluorescence and photosynthetic parameters of *B*.*cusia*. F0: minimal fluorescence; Fm: maximal fluorescence; Fv: variable fluorescence; GS: stomatal conductance; CI: intercellular CO_2_ concentration; EVPA: transpiration rate; PN: net photosynthetic rate and PAR: photosynthetically active radiation.

MeJA stress severely affected gas exchange parameters and chlorophyll fluorescence compared to the control (Fig 7). Chlorophyll fluorescence of leaves treated with methyl jasmonate was significantly decreased, with the minimal fluorescence (F0), maximal fluorescence (Fm), variable fluorescence (Fv), Fv/F0 and Fv/Fm values of TR all lower than those of CR (*p* = 0.000, 0.000, 0.000, 0.016, 0.009, respectively). The leaves receiving MeJA showed lower photosynthesis compared to the control in the absence of MeJA. Leaves of *B*. *cusia* receiving 22.29 μM MeJA showed an increase in GS by 215.60%, in CI by 12.62% and in EVPA by 142.34%, but a decrease in PN by 51.58% in comparison with the control. However, no obvious increase of PAR was observed compared to the control. In general, the transpiration of leaves showed a negative correlation with photosynthesis. Leaves of *B*. *cusia* treated by methyl jasmonate enhanced the transpiration rate and indirectly inhibited the photosynthesis of leaves.

## Discussion

From Fig 3, we can see that Erythrose 4-phosphate is a precursor in tryptophan biosynthesis, which is an intermediate in the pentose phosphate pathway and the Calvin cycle. The Calvin cycle is a series of chemical reactions occurring in the chloroplast during photosynthesis. Photosynthesis affects the production of Erythrose 4-phosphate, which in turn affects the biosynthesis of tryptophan. Therefore, this study measured photosynthesis to demonstrate that exogenous methyl jasmonate may affect the synthesis of tryptophan through photosynthesis. In this study, chlorophyll fluorescence measurements show that leaves’ photosynthesis was reduced in the leaves, and that photosynthesis was suppressed after treatment with 22.29 μM MeJA, and DEGs of CL-VS-TL involved in “photosystem”, “photosynthetic membrane”, “photosynthesis-antenna proteins” were down-regulated, which further verified that MeJA induce leaf senescence in *B*. *cusia*. The appearance and traits of the leaves and roots treated with different concentrations of MeJA showed that the leaf areas decreased and the numbers of hair roots increased. These results were consistent with previous reports about the effects of MeJA on leaf senescence [41–43] and lateral roots formation [44–47]. The effects of different concentrations of methyl jasmonate on *Baphicacanthus cusia* (Nees) Bremek in different ecological environments were further studied in the future.

RNA-seq has been applied to gene expression analysis on the genome-wide level in a number of plants [48–50]. In a previous study [51], qRT-PCR assays were employed to analyze the expression stability of ten candidate reference genes and the expression levels of two genes involved in synthesis of terpenoid indole alkaloids of *B*. *cusia* after 24 h MeJA treatment; However, the qRT-PCR assay is limited by the number of sequencing fragments. The RNA-seq method allows for comprehensive and accurate quantitative information on gene expression to acquire better gene regulatory profiles and to identify more DEGs. In this study, we obtained 33,317 annotated unigenes and analyzed 8,355 DEG expression levels, especially genes related to tryptophan synthesis. The results revealed that the relative expression of all the DEGs involved in tryptophan synthesis were down-regulated in leaves treated by MeJA after seven days. However, the relative expression levels of aroF, aroK, trpD and trp3 were up-regulated in roots, which is inconsistent with the results of the previous study [51].

After treatment with methyl jasmonate, the indigo content of the leaves and roots from treated *B*. *cusia* increased. This was consistent with the results of the previous study [51]. However, expression levels of the eight related genes involved in phenylalanine, tyrosine and tryptophan biosynthesis were down-regulated in leaves and gene expression levels of aroA were down-regulated, while the other seven genes were up-regulated in roots of *B*. *cusia* treated by MeJA. This indicates that exogenous MeJA regulates the tryptophan biosynthetic pathway, which further affects the expression of downstream genes involved in indican biosynthesis. Genes involve in the indican metabolism pathway, which is upstream of indigo and indirubin biosynthesis, may also be induced by MeJA treatment; such genes include cytochrome P450, UDP-glycosyltransferase, glucosidase and tryptophan synthase [40]. Our data provide a valuable resource for discovering candidate genes related to indigo, indirubin and indican biosynthesis in response to MeJA, especially TrpA. TrpA is a heterodimeric enzyme with two α and β subunits. The α-subunit catalyzes indole production and the β-subunit catalyzes tryptophan yield [52, 53]. In this study, expression levels of TrpA β-subunits were down-regulated in leaves, which would increase indole biosynthesis. Hence, DEGs of trpA β-subunit may be the key upstream genes leading to the production of indigo in leaves of *B*. *cusia*. However, unlike leaves, DEGs of trp3 may be the key upstream genes leading to the production of indigo in roots of *B*. *cusia*.

The optimal defense theory (ODT) and the growth-differentiation balance hypothesis (GDBH) were two excellent theories that attempted to explain the expression patterns of chemical defense in plants. The ODT proposed that high fitness value plant parts would be highly defended, but the GDBH speculated that slow-growing plant parts would be highly defended [54]. Tryptophan acts as a biochemical precursor of auxin, which plays a key role in the plant life cycle and development [55]. In this study, we found that DEGs related to growth and development processes were down-regulated in the treated leaves, such as “photosystem”, “photosynthesis-antenna proteins”, catalytic activity and “negative regulation of biological process”, while most of the unigenes involved in the defense response were up-regulated in treated roots, such as “catalytic activity”, “carbohydrate metabolic process”, “sesquiterpenoid and triterpenoid biosynthesis”, and oxidoreductase activity. Similar findings from GO and KEGG functional annotation support the view of JAs playing a key role in regulating resource distribution between the defense and growth competition processes [56]. These results support established theories of ODT [57, 58]. This growth-defense trade-off may help *B*. *cusia* improve its adaptability by shifting the energy conservation of down-regulated photosynthesis to a relevant defense response in a changing environment.

## Materials and methods

### Plant materials and MeJA treatment

The *Baphicacanthus cusia* (Nees) Bremek (*B*. *cusia*) samples were collected from our experimental field at the Fujian Agriculture and Forestry University (26.0822, 119.2398). The *B*. *cusia* samples were propagated from cuttings and planted in our experimental field. When shoots were rooted and well-established, seedlings were selected from the experimental field and planted into shade plots. All leaves of treated groups (TL: Treated Leaf and TR: Treated Root) were sprayed with a solution of 0.01% (v/v) Tween 20 containing 22.29 μM MeJA (Sigma-Aldrich), and the leaves of control groups (CL: Controlled Leaf and CR: Controlled Root) were sprayed with 0.01% (v/v) Tween 20 without MeJA to the point of runoff. After treatment, the second to fourth leaves from the top of plants were harvested for physicochemical and molecular analysis. The collected leaves and roots were frozen in liquid nitrogen, and then stored at -80 °C for future analyses. Three biological replicates from the independent control and treated *B*. *cusia* were prepared for RNA sequencing. Each biological replicate used three root or leaf plants. qRT-PCR was performed using three biological and three technical replicates.

### RNA isolation, sequencing, assembly and bioinformatics analysis

Total RNA was isolated with the EASYspin plant RNA kit (Aidlab Ltd, Beijing, China). RNA purity was validated using a Biodrop spectrophotometer (Biochrom Ltd). RNA integrity was analyzed using Agilent Bioanalyzer 2100 (Agilent Technologies). The construction of the RNA library and RNA sequencing of the libraries were performed by commercial service providers Gene denovo Biotechnology Co. (Guangzhou, China) under the Illumina HiSeq 4000. The RNA-seq data of the treated-by-MeJA and control *B*. *cusia* were deposited in the NCBI SRA repository: SRA628524 (SRP124081: PRJNA415260). The raw reads were processed and analyzed using the method previously reported [40]. The assembled unigenes were BLASTX searched and annotated against the databases of NCBI non-redundant protein (NR), SwissProt, euKaryotic Orthologous Groups (KOG), Gene Ontology (GO), and Kyoto Encyclopedia of Genes and Genomes (KEGG) with an E-value threshold of 1E-5. The gene abundances were calculated and normalized to reads per kb per million reads (RPKM). The significant DEGs were identified with the threshold of fold change >2 and false discovery rate (FDR) <0.05. The calculated *p*-value was obtained by FDR correction, and the *q*-value is the multi-hypothesis test corrected *p*-value.

### Validation of qRT-PCR

Expression levels of the eight candidate genes, aroF, aroK, aroA, aroC, trp3, trpD, trpA-α and trpA-β were evaluated; these genes are involved in phenylalanine, tyrosine and trytophan biosynthesis. Primers of the eight candidate genes and glyceraldehyde-3-phosphate dehydrogenase (GAPDH) used for the reference gene were designed to amplify short regions using primer 3 web (version 4.1.0, http://bioinfo.ut.ee/primer3/, S5 Table). RNA was reverse transcribed using the Fast Quantity RT Kit (TIANGEN, China) according to the manufacturer’s specifications. qRT-PCR was carried out by using the SYBR Premix Ex Taq kit (TaKaRa Bio Inc). The amplification was executed with the following PCR program: 90 s at 95 °C, 40 cycles of 5 s at 95 °C for denaturation, 15 s at 60 °C for annealing, 20 s at 72 °C for elongation, and 65°C~90 °C for melting curve analysis. qRT-PCR was performed on the ABI PRISM 7500 Real-Time PCR System (Applied Biosystems, US). The relative expression ratios of each candidate gene were calculated based on the comparative CT (2^−ΔΔCt^) method.

### Analysis with high-performance liquid chromatography

The HPLC analysis was carried out using an LC-20AT HPLC system (Shimadzu, Japan). Methanol and water, with a volume ratio of 70: 30 was used as the mobile phase for both indigo and indirubin, and the flow rate was 1.0 ml/min. To prepare the solutions, samples of the leaves and roots were extracted by a Soxhlet extractor (frequency of 40 KHz, power of 500 W). The filtrates were combined and swept in a rotary evaporator. The mixed reference solutions of indigo and indirubin were dissolved in N, N-dimethyl formamide in triplicate. After filtrated with a 0.45 μm filter, 10 μl of sample solutions were injected into HPLC in duplicate.

### Measurement of photosynthetic characteristics and chlorophyll fluorescence

The chlorophyll fluorescence of minimal fluorescence (F0), maximal fluorescence (Fm), variable fluorescence (Fv), Fv/F0 and Fv/Fm were measured in fully expanded leaves of *B*. *cusia* using FluorCam6 Fluorometer (Photon Systems Instruments, Czech Republic). The gas exchange parameters of stomatal conductance (GS), intercellular CO_2_ concentration (CI), transpiration rate (EVPA), net photosynthetic rate (PN), and photosynthetically active radiation (PAR) were measured using the portable photosynthesis system CIRAS-2 (Hansatech, UK). All determinations were repeated in triplicates. The measurements were carried out at 10 am in sunny weather.

## Supporting information

S1 Table Pathway annotation of significant difference gene between CL and TL. (XLS)

S2 Table Pathway annotation of significant difference gene between CR and TR. (XLS)

S3 Table Significant enrichment analysis of GO function between CL and TL. (XLS)

S4 Table Significant enrichment analysis of GO function between CR and TR. (XLS)

S5 Table Primer sequences of eight candidate genes and GAPDH used for qRT-PCR. (XLSX)

S6 Table The growth conditions for field trials. (XLS)

S7 Table Leaves length and width of *B*. *cusia* treated with MeJA. (XLSX)

S1 Fig map00195 of CL-VS-TL. (TIF)

S2 Fig map00196 of CL-VS-TL. (TIF)

S3 Fig map00195 of CR-VS-TR. (TIF)

S4 Fig map00196 of CR-VS-TR. (TIF)

S5 Fig Original group figures of different concentrations of MeJA on *B*. *cusia* leaves and roots. (TIF)

S6 Fig Original and magnified figures of different concentrations of MeJA on *B*. *cusia* leaves and roots. (TIF)

## References

[1] Kooyers NJ, Blackman BK, Holeski LM. Optimal defense theory explains deviations from latitudinal herbivory defense hypothesis. Ecology. 2017;98(4):1036–48. Epub 2017/01/12. 10.1002/ecy.1731 . 28074474

[2] Biere A, Marak HB, van Damme JM. Plant chemical defense against herbivores and pathogens: generalized defense or trade-offs? Oecologia. 2004;140(3):430–41. Epub 2004/05/18. 10.1007/s00442-004-1603-6 . 15146326

[3] Mitra S, Baldwin IT. RuBPCase activase (RCA) mediates growth-defense trade-offs: silencing RCA redirects jasmonic acid (JA) flux from JA-isoleucine to methyl jasmonate (MeJA) to attenuate induced defense responses in Nicotiana attenuata. New Phytol. 2014;201(4):1385–95. Epub 2014/02/05. 10.1111/nph.12591 24491116 PMC4996320

[4] Caarls L, Elberse J, Awwanah M, Ludwig NR, de Vries M, Zeilmaker T, et al Arabidopsis JASMONATE-INDUCED OXYGENASES down-regulate plant immunity by hydroxylation and inactivation of the hormone jasmonic acid. Proc Natl Acad Sci U S A. 2017;114(24):6388–93. Epub 2017/06/01. 10.1073/pnas.1701101114 28559313 PMC5474790

[5] Liu JJ, Williams H, Li XR, Schoettle AW, Sniezko RA, Murray M, et al Profiling methyl jasmonate-responsive transcriptome for understanding induced systemic resistance in whitebark pine (Pinus albicaulis). Plant Mol Biol. 2017;95(4–5):359–74. Epub 2017/09/02. 10.1007/s11103-017-0655-z . 28861810

[6] Huang H, Liu B, Liu L, Song S. Jasmonate action in plant growth and development. J Exp Bot. 2017;68(6):1349–59. Epub 2017/02/06. 10.1093/jxb/erw495 . 28158849

[7] Kikuchi M, Ueno M, Itoh Y, Suda W, Hattori M. Uremic Toxin-Producing Gut Microbiota in Rats with Chronic Kidney Disease. Nephron. 2017;135(1):51–60. Epub 2016/10/05. 10.1159/000450619 . 27701177

[8] Hu Y, Jiang Y, Han X, Wang H, Pan J, Yu D. Jasmonate regulates leaf senescence and tolerance to cold stress: crosstalk with other phytohormones. J Exp Bot. 2017;68(6):1361–9. Epub 2017/02/16. 10.1093/jxb/erx004 . 28201612

[9] Jeon BJ, Yang HM, Lyu YS, Pae HO, Ju SM, Jeon BH. Apigenin inhibits indoxyl sulfate-induced endoplasmic reticulum stress and anti-proliferative pathways, CHOP and IL-6/p21, in human renal proximal tubular cells. Eur Rev Med Pharmacol Sci. 2015;19(12):2303–10. Epub 2015/07/15. . 26166660

[10] Wong J, Piceno YM, DeSantis TZ, Pahl M, Andersen GL, Vaziri ND. Expansion of urease- and uricase-containing, indole- and p-cresol-forming and contraction of short-chain fatty acid-producing intestinal microbiota in ESRD. Am J Nephrol. 2014;39(3):230–7. Epub 2014/03/20. 10.1159/000360010 24643131 PMC4049264

[11] Rahnamaie-Tajadod R, Loke KK, Goh HH, Noor NM. Differential Gene Expression Analysis in Polygonum minus Leaf upon 24 h of Methyl Jasmonate Elicitation. Front Plant Sci. 2017;8:109 Epub 2017/02/22. 10.3389/fpls.2017.00109 28220135 PMC5292430

[12] Niwa T. Targeting protein-bound uremic toxins in chronic kidney disease. Expert Opin Ther Targets. 2013;17(11):1287–301. Epub 2013/08/15. 10.1517/14728222.2013.829456 . 23941498

[13] Schulman G. A nexus of progression of chronic kidney disease: tryptophan, profibrotic cytokines, and charcoal. J Ren Nutr. 2012;22(1):107–13. Epub 2011/12/28. 10.1053/j.jrn.2011.10.035 . 22200426

[14] Lee CT, Lee YT, Ng HY, Chiou TT, Cheng CI, Kuo CC, et al Lack of modulatory effect of simvastatin on indoxyl sulfate-induced activation of cultured endothelial cells. Life Sci. 2012;90(1–2):47–53. Epub 2011/11/08. 10.1016/j.lfs.2011.10.014 . 22056374

[15] Chiang CK, Tanaka T, Inagi R, Fujita T, Nangaku M. Indoxyl sulfate, a representative uremic toxin, suppresses erythropoietin production in a HIF-dependent manner. Lab Invest. 2011;91(11):1564–71. Epub 2011/08/25. 10.1038/labinvest.2011.114 . 21863063

[16] Jeong YJ, An CH, Park SC, Pyun JW, Lee J, Kim SW, et al Methyl Jasmonate Increases Isoflavone Production in Soybean Cell Cultures by Activating Structural Genes Involved in Isoflavonoid Biosynthesis. J Agric Food Chem. 2018;66(16):4099–105. Epub 2018/04/10. 10.1021/acs.jafc.8b00350 . 29630360

[17] Deng Y, Li C, Li H, Lu S. Identification and Characterization of Flavonoid Biosynthetic Enzyme Genes in Salvia miltiorrhiza (Lamiaceae). Molecules. 2018;23(6). Epub 2018/06/20. 10.3390/molecules23061467 . 29914175 PMC6099592

[18] Delgado LD, Zuniga PE, Figueroa NE, Pastene E, Escobar-Sepulveda HF, Figueroa PM, et al Application of a JA-Ile Biosynthesis Inhibitor to Methyl Jasmonate-Treated Strawberry Fruit Induces Upregulation of Specific MBW Complex-Related Genes and Accumulation of Proanthocyanidins. Molecules. 2018;23(6). Epub 2018/06/15. 10.3390/molecules23061433 . 29899259 PMC6100305

[19] Taki K, Takayama F, Niwa T. Beneficial effects of Bifidobacteria in a gastroresistant seamless capsule on hyperhomocysteinemia in hemodialysis patients. J Ren Nutr. 2005;15(1):77–80. Epub 2005/01/14. . 15648012 10.1053/j.jrn.2004.09.028

[20] Miyazaki T, Ise M, Hirata M, Endo K, Ito Y, Seo H, et al Indoxyl sulfate stimulates renal synthesis of transforming growth factor-beta 1 and progression of renal failure. Kidney Int Suppl. 1997;63:S211–4. Epub 1998/01/04. . 9407462

[21] Minami Y, Takao H, Kanafuji T, Miura K, Kondo M, Hara-Nishimura I, et al beta-Glucosidase in the indigo plant: intracellular localization and tissue specific expression in leaves. Plant Cell Physiol. 1997;38(9):1069–74. Epub 1997/11/14. . 9360324 10.1093/oxfordjournals.pcp.a029273

[22] Liu H, Meng F, Miao H, Chen S, Yin T, Hu S, et al Effects of postharvest methyl jasmonate treatment on main health-promoting components and volatile organic compounds in cherry tomato fruits. Food Chem. 2018;263:194–200. Epub 2018/05/23. 10.1016/j.foodchem.2018.04.124 . 29784307

[23] D’Onofrio C, Matarese F, Cuzzola A. Effect of methyl jasmonate on the aroma of Sangiovese grapes and wines. Food Chem. 2018;242:352–61. Epub 2017/10/19. 10.1016/j.foodchem.2017.09.084 . 29037700

[24] Chen H, Shao J, Zhang H, Jiang M, Huang L, Zhang Z, et al Sequencing and Analysis of Strobilanthes cusia (Nees) Kuntze Chloroplast Genome Revealed the Rare Simultaneous Contraction and Expansion of the Inverted Repeat Region in Angiosperm. Front Plant Sci. 2018;9:324 Epub 2018/03/30. 10.3389/fpls.2018.00324 29593773 PMC5861152

[25] Zhou B, Yang Z, Feng Q, Liang X, Li J, Zanin M, et al Aurantiamide acetate from baphicacanthus cusia root exhibits anti-inflammatory and anti-viral effects via inhibition of the NF-kappaB signaling pathway in Influenza A virus-infected cells. J Ethnopharmacol. 2017;199:60–7. Epub 2017/01/26. 10.1016/j.jep.2017.01.038 . 28119097

[26] Gu W, Zhang Y, Hao XJ, Yang FM, Sun QY, Morris-Natschke SL, et al Indole alkaloid glycosides from the aerial parts of Strobilanthes cusia. J Nat Prod. 2014;77(12):2590–4. Epub 2014/11/27. 10.1021/np5003274 . 25427242

[27] Feng QT, Zhu GY, Gao WN, Yang Z, Zhong N, Wang JR, et al Two New Alkaloids from the Roots of Baphicacanthus cusia. Chem Pharm Bull (Tokyo). 2016;64(10):1505–8. Epub 2016/10/12. 10.1248/cpb.c16-00315 . 27725504

[28] Charoenchai P, Vajrodaya S, Somprasong W, Mahidol C, Ruchirawat S, Kittakoop P. Part 1: Antiplasmodial, cytotoxic, radical scavenging and antioxidant activities of Thai plants in the family Acanthaceae. Planta Med. 2010;76(16):1940–3. Epub 2010/06/18. 10.1055/s-0030-1250045 . 20556707

[29] Tanaka T, Ikeda T, Kaku M, Zhu XH, Okawa M, Yokomizo K, et al A new lignan glycoside and phenylethanoid glycosides from Strobilanthes cusia BREMEK. Chem Pharm Bull (Tokyo). 2004;52(10):1242–5. Epub 2004/10/07. . 15467245 10.1248/cpb.52.1242

[30] Gu W, Wang W, Li XN, Zhang Y, Wang LP, Yuan CM, et al A novel isocoumarin with anti-influenza virus activity from Strobilanthes cusia. Fitoterapia. 2015;107:60–2. Epub 2015/10/28. 10.1016/j.fitote.2015.10.009 . 26506123

[31] Mak NK, Leung CY, Wei XY, Shen XL, Wong RN, Leung KN, et al Inhibition of RANTES expression by indirubin in influenza virus-infected human bronchial epithelial cells. Biochem Pharmacol. 2004;67(1):167–74. Epub 2003/12/12. . 14667939 10.1016/j.bcp.2003.08.020

[32] Ko HC, Wei BL, Chiou WF. The effect of medicinal plants used in Chinese folk medicine on RANTES secretion by virus-infected human epithelial cells. J Ethnopharmacol. 2006;107(2):205–10. Epub 2006/04/20. 10.1016/j.jep.2006.03.004 . 16621378

[33] Ho YL, Kao KC, Tsai HY, Chueh FY, Chang YS. Evaluation of antinociceptive, anti-inflammatory and antipyretic effects of Strobilanthes cusia leaf extract in male mice and rats. Am J Chin Med. 2003;31(1):61–9. Epub 2003/05/02. 10.1142/S0192415X03000783 . 12723755

[34] Zhu HH, Wu DP, Du X, Zhang X, Liu L, Ma J, et al Oral arsenic plus retinoic acid versus intravenous arsenic plus retinoic acid for non-high-risk acute promyelocytic leukaemia: a non-inferiority, randomised phase 3 trial. Lancet Oncol. 2018 Epub 2018/06/10. 10.1016/S1470-2045(18)30295-X . 29884593

[35] Zhu HH, Wu DP, Jin J, Li JY, Ma J, Wang JX, et al Oral tetra-arsenic tetra-sulfide formula versus intravenous arsenic trioxide as first-line treatment of acute promyelocytic leukemia: a multicenter randomized controlled trial. J Clin Oncol. 2013;31(33):4215–21. Epub 2013/10/16. 10.1200/JCO.2013.48.8312 . 24127444

[36] Naganuma M, Sugimoto S, Mitsuyama K, Kobayashi T, Yoshimura N, Ohi H, et al Efficacy of Indigo Naturalis in a Multicenter Randomized Controlled Trial of Patients With Ulcerative Colitis. Gastroenterology. 2018;154(4):935–47. Epub 2017/11/28. 10.1053/j.gastro.2017.11.024 . 29174928

[37] Kawai S, Iijima H, Shinzaki S, Hiyama S, Yamaguchi T, Araki M, et al Indigo Naturalis ameliorates murine dextran sodium sulfate-induced colitis via aryl hydrocarbon receptor activation. J Gastroenterol. 2017;52(8):904–19. Epub 2016/12/03. 10.1007/s00535-016-1292-z . 27900483

[38] Lin YK, See LC, Huang YH, Chi CC, Hui RC. Comparison of indirubin concentrations in indigo naturalis ointment for psoriasis treatment: a randomized, double-blind, dosage-controlled trial. Br J Dermatol. 2018;178(1):124–31. Epub 2017/08/18. 10.1111/bjd.15894 . 28815560

[39] Liau BC, Jong TT, Lee MR, Chen SS. LC-APCI-MS method for detection and analysis of tryptanthrin, indigo, and indirubin in daqingye and banlangen. J Pharm Biomed Anal. 2007;43(1):346–51. Epub 2006/08/04. 10.1016/j.jpba.2006.06.029 16884885 PMC7126482

[40] Lin W, Huang W, Ning S, Wang X, Ye Q, Wei D. De novo characterization of the Baphicacanthus cusia(Nees) Bremek transcriptome and analysis of candidate genes involved in indican biosynthesis and metabolism. Plos One. 2018;13(7):e0199788 Epub 2018/07/06. 10.1371/journal.pone.0199788 . 29975733 PMC6033399

[41] Tan XL, Fan ZQ, Shan W, Yin XR, Kuang JF, Lu WJ, et al Association of BrERF72 with methyl jasmonate-induced leaf senescence of Chinese flowering cabbage through activating JA biosynthesis-related genes. Hortic Res. 2018;5:22 Epub 2018/05/08. 10.1038/s41438-018-0028-z 29736247 PMC5928098

[42] Ji Y, Liu J, Xing D. Low concentrations of salicylic acid delay methyl jasmonate-induced leaf senescence by up-regulating nitric oxide synthase activity. J Exp Bot. 2016;67(17):5233–45. Epub 2016/07/22. 10.1093/jxb/erw280 . 27440938

[43] Zhang Y, Liu J, Chai J, Xing D. Mitogen-activated protein kinase 6 mediates nuclear translocation of ORE3 to promote ORE9 gene expression in methyl jasmonate-induced leaf senescence. J Exp Bot. 2016;67(1):83–94. Epub 2015/10/29. 10.1093/jxb/erv438 . 26507893

[44] Kuo CH. Role of rice heme oxygenase in lateral root formation. Plant Signal Behav. 2013;8(10): Epub 2013/07/28. 23887491 10.4161/psb.25766 PMC4091076

[45] Sun J, Chen Q, Qi L, Jiang H, Li S, Xu Y, et al Jasmonate modulates endocytosis and plasma membrane accumulation of the Arabidopsis PIN2 protein. New Phytol. 2011;191(2):360–75. Epub 2011/04/07. 10.1111/j.1469-8137.2011.03713.x . 21466556

[46] Sun J, Xu Y, Ye S, Jiang H, Chen Q, Liu F, et al Arabidopsis ASA1 is important for jasmonate-mediated regulation of auxin biosynthesis and transport during lateral root formation. Plant Cell. 2009;21(5):1495–511. Epub 2009/05/14. 10.1105/tpc.108.064303 19435934 PMC2700526

[47] Xue R, Zhang B. Increased endogenous methyl jasmonate altered leaf and root development in transgenic soybean plants. J Genet Genomics. 2007;34(4):339–46. Epub 2007/05/15. 10.1016/S1673-8527(07)60036-8 . 17498632

[48] Ciura J, Szeliga M, Grzesik M, Tyrka M. Changes in fenugreek transcriptome induced by methyl jasmonate and steroid precursors revealed by RNA-Seq. Genomics. 2017 Epub 2017/11/07. 10.1016/j.ygeno.2017.10.006 . 29107013

[49] Shi J, Ma C, Qi D, Lv H, Yang T, Peng Q, et al Transcriptional responses and flavor volatiles biosynthesis in methyl jasmonate-treated tea leaves. Bmc Plant Biol. 2015;15:233 Epub 2015/10/01. 10.1186/s12870-015-0609-z 26420557 PMC4588909

[50] Li ST, Zhang P, Zhang M, Fu CH, Zhao CF, Dong YS, et al Transcriptional profile of Taxus chinensis cells in response to methyl jasmonate. Bmc Genomics. 2012;13:295 Epub 2012/07/04. 10.1186/1471-2164-13-295 22748077 PMC3414795

[51] Huang Y, Tan H, Yu J, Chen Y, Guo Z, Wang G, et al Stable Internal Reference Genes for Normalizing Real-Time Quantitative PCR in Baphicacanthus cusia under Hormonal Stimuli and UV Irradiation, and in Different Plant Organs. Front Plant Sci. 2017;8:668 Epub 2017/05/19. 10.3389/fpls.2017.00668 28515733 PMC5413499

[52] Loutchko D, Gonze D, Mikhailov AS. Single-Molecule Stochastic Analysis of Channeling Enzyme Tryptophan Synthase. J Phys Chem B. 2016;120(9):2179–86. Epub 2016/02/11. 10.1021/acs.jpcb.5b12229 . 26863529

[53] Radwanski ER, Last RL. Tryptophan biosynthesis and metabolism: biochemical and molecular genetics. Plant Cell. 1995;7(7):921–34. Epub 1995/07/01. 10.1105/tpc.7.7.921 7640526 PMC160888

[54] Barto EK, Cipollini D. Testing the optimal defense theory and the growth-differentiation balance hypothesis in Arabidopsis thaliana. Oecologia. 2005;146(2):169–78. Epub 2005/08/13. 10.1007/s00442-005-0207-0 . 16096848

[55] Bai B, Novak O, Ljung K, Hanson J, Bentsink L. Combined transcriptome and translatome analyses reveal a role for tryptophan-dependent auxin biosynthesis in the control of DOG1-dependent seed dormancy. New Phytol. 2018;217(3):1077–85. Epub 2017/11/16. 10.1111/nph.14885 . 29139127

[56] Attaran E, Major IT, Cruz JA, Rosa BA, Koo AJ, Chen J, et al Temporal Dynamics of Growth and Photosynthesis Suppression in Response to Jasmonate Signaling. Plant Physiol. 2014;165(3):1302–14. Epub 2014/05/14. 10.1104/pp.114.239004 24820026 PMC4081338

[57] Heath JJ, Kessler A, Woebbe E, Cipollini D, Stireman JO 3rd. Exploring plant defense theory in tall goldenrod, Solidago altissima. New Phytol. 2014;202(4):1357–70. Epub 2014/03/13. 10.1111/nph.12755 . 24611577

[58] Rojas CM, Senthil-Kumar M, Tzin V, Mysore KS. Regulation of primary plant metabolism during plant-pathogen interactions and its contribution to plant defense. Front Plant Sci. 2014;5:17 Epub 2014/02/28. 10.3389/fpls.2014.00017 24575102 PMC3919437

