# Supplementary figures and images for: Comparative transcriptome analyses revealed differential strategies of roots and leaves from methyl jasmonate treatment Baphicacanthus cusia (Nees) Bremek and differentially expressed genes involved in tryptophan biosynthesis
Source: PLoS One. 2019 Mar 13;14(3):e0212863. doi: 10.1371/journal.pone.0212863 (PMC6415880; doi:10.1371/journal.pone.0212863)

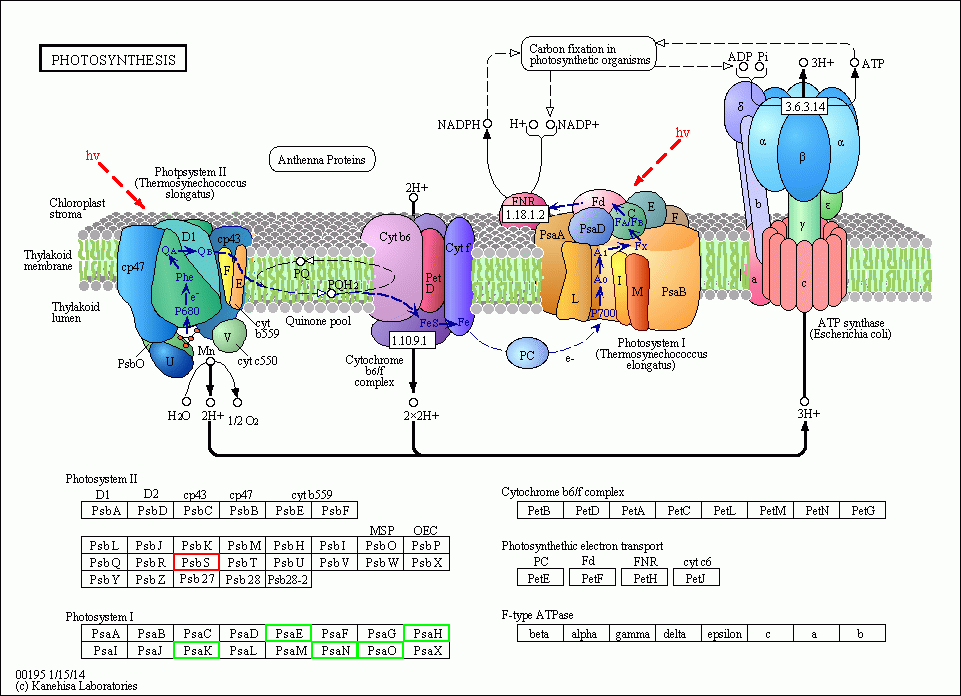

Supplement: S1 Fig — (TIF) [file pone.0212863.s008.tif]

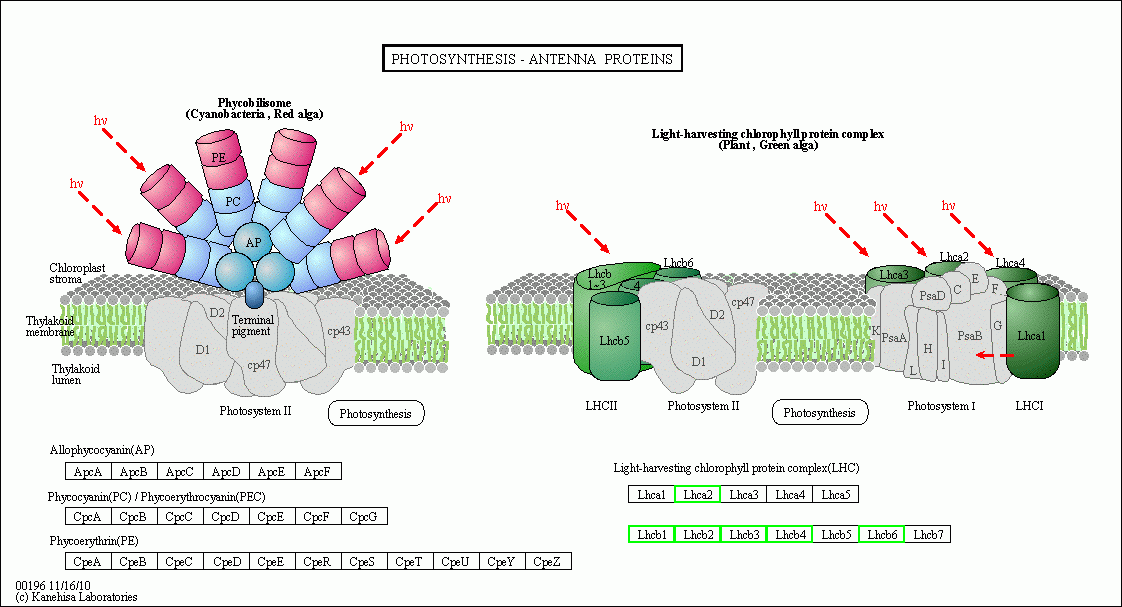

Supplement: S2 Fig — (TIF) [file pone.0212863.s009.tif]

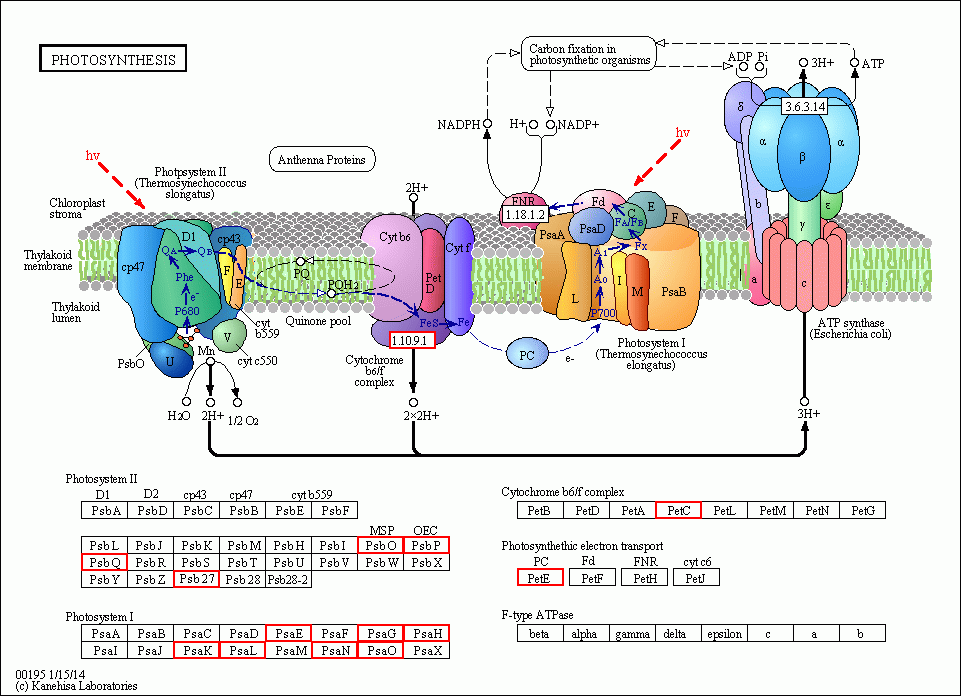

Supplement: S3 Fig — (TIF) [file pone.0212863.s010.tif]

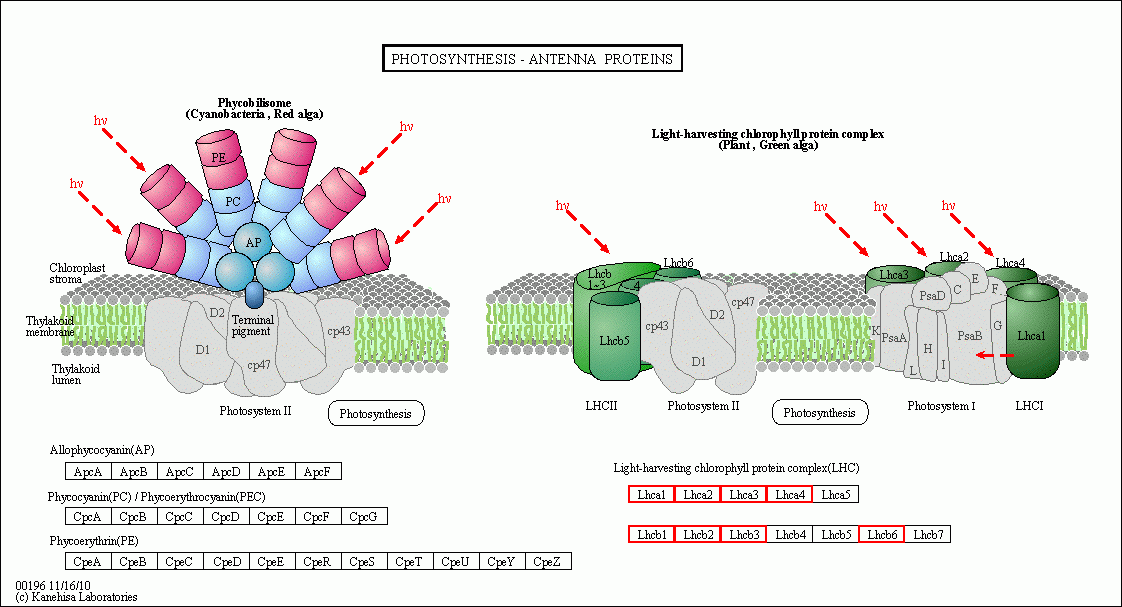

Supplement: S4 Fig — (TIF) [file pone.0212863.s011.tif]

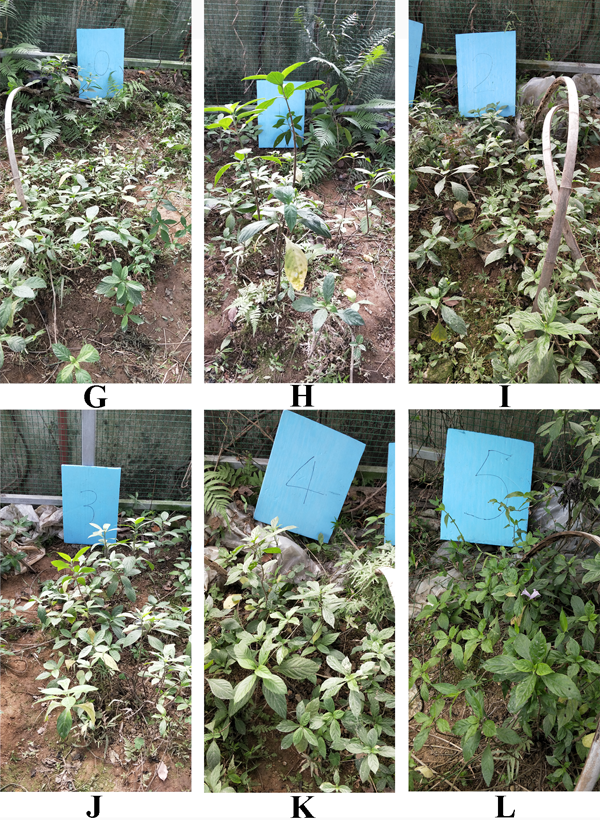

Supplement: S5 Fig — (TIF) [file pone.0212863.s012.tif]

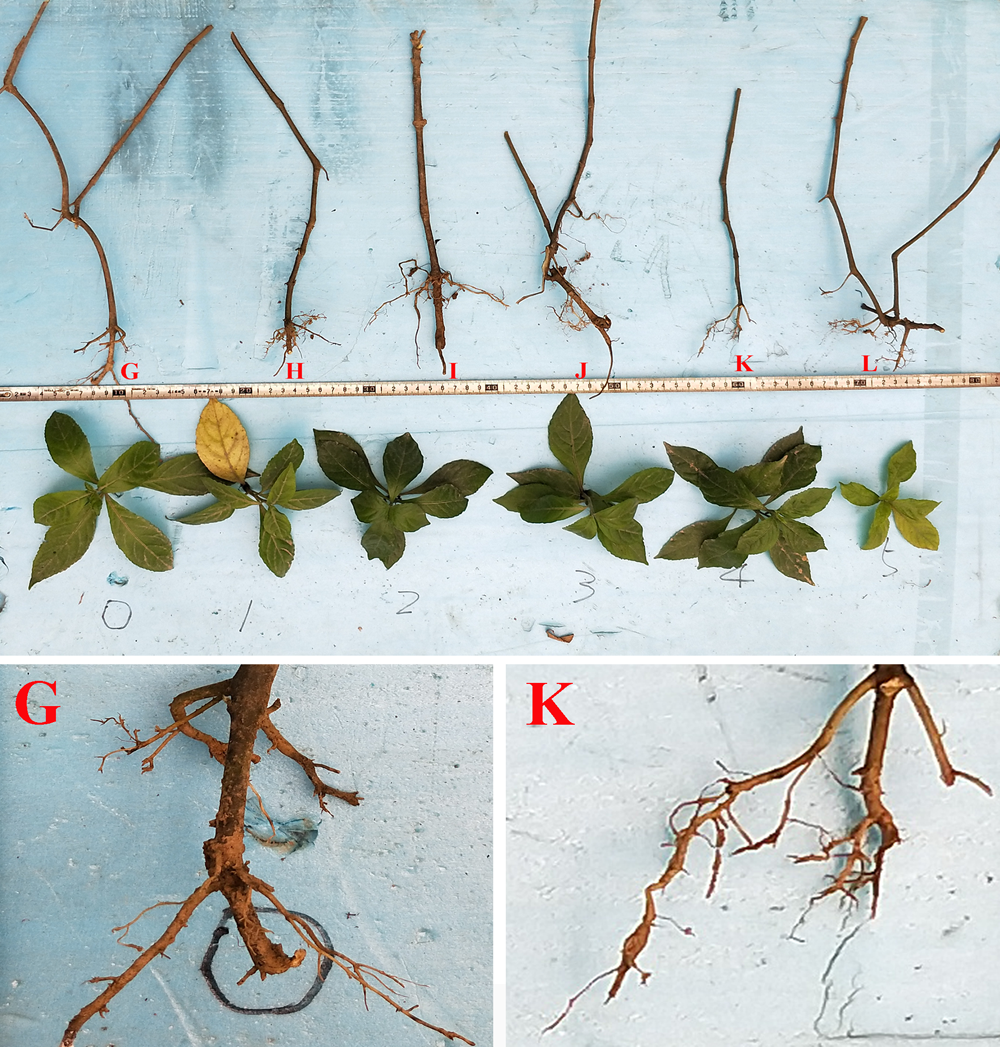

Supplement: S6 Fig — (TIF) [file pone.0212863.s013.tif]
